# Investigating the Effects of Yunnan Lufeng Aromatic Vinegar Intervention on Intestinal Microbiota, SCFAs, and Metabolites in Mice Using Multi-Omics Techniques

**DOI:** 10.3390/foods14213747

**Published:** 2025-10-31

**Authors:** Hongqin Chen, Ruihuan Zhao, Zhichao Xiao, Yang Li, Junran Yang, Shuaihan Jiang, Sisi Xu, Zhiqiang Xu, Dahai Gu

**Affiliations:** 1 College of Food Science and Technology, Yunnan Agricultural University, Kunming 650201, China; 2 College of Health, Yunnan Technology and Business University, Kunming 651701, China

**Keywords:** Yunnan Lufeng aromatic vinegar, gut microbiota, short-chain fatty acids, untargeted metabolomics

## Abstract

This study systematically assessed the regulatory effects of Yunnan Lufeng aromatic vinegar (LFAV) on the intestinal microbiota composition, short-chain fatty acids, and cecal metabolites in mice. 16S rRNA high-throughput sequencing revealed that LFAV intervention significantly altered gut microbiota diversity; the M-L group exhibited 19.98% unique operational taxonomic units, while both Chao1 (496.63 ± 42.14) and Shannon indices (6.68 ± 0.32) increased by 37.46% and 3.25%, respectively, compared to the blank group, indicating enhanced microbiota richness. Species composition analysis demonstrated that the relative abundance of Firmicutes reached 75.4% in the M-L group, a 24.4% increase over the B group, whereas Bacteroidetes abundance decreased to 8.2%. GC-MS analysis detected peak butyric acid levels in the M-L group. Untargeted metabolomics identified 520 metabolites, of which 60 were significant differential metabolites. Cluster heatmap and Z-score analyses demonstrated that LFAV intervention significantly modulated mouse metabolites. KEGG pathway enrichment analysis indicated the upregulation of pathways including neuroactive ligand–receptor interactions and renin secretion. Pearson correlation analysis showed a strong positive correlation (*p* < 0.01) between Lactobacillus and acetic acid/butyric acid; concurrently, increased Lactobacillus proliferation and elevated butyric acid levels were observed in the M-L and M-M groups. These findings suggest that LFAV intervention promotes the proliferation of beneficial bacteria, which may improve intestinal health. Collectively, LFAV significantly modified gut microbiota structure and metabolites in mice, highlighting its potential as a natural prebiotic or functional food ingredient and providing a scientific basis for developing functional vinegar products.

## 1. Introduction

Vinegar is a traditional fermented product with a global consumption history exceeding 3000 years [1]. As a liquid foodstuff typically produced from cereals or fruits via microbial fermentation, vinegar serves widely as an acidic condiment and also functions as a preservative within the food industry [2]. Beyond its culinary applications, vinegar possesses recognized health-promoting and medicinal properties, such as reducing cholesterol and blood pressure, exhibiting antibacterial activity, aiding digestion, and offering hepatoprotective effects [3]. Lufeng aromatic vinegar (LFAV), a fermented rice vinegar originating from Lufeng County, Chuxiong City, Yunnan Province, China, is manufactured using glutinous rice and involves saccharification, alcoholic fermentation, and acetic acid fermentation stages facilitated by yeast, rice koji, red yeast rice, and other starters [4]. With origins dating to the Ming Dynasty (1621), LFAV boasts a nearly 400-year history and is a renowned regional product in Yunnan, achieving designation as a National Geographical Indication Protected Product in 2015. Current research on LFAV primarily focuses on flavor characterization and process optimization. Zhao et al. (2024) explored bacterial diversity across different aging periods, revealing significant variations in bacterial community composition; samples from 2017, 2018, and 2019 exhibited higher diversity, dominated by *Firmicutes*, *Proteobacteria*, and *Actinobacteriota* [4]. Furthermore, Zhao et al. (2023) employed headspace solid-phase microextraction coupled with gas chromatography–mass spectrometry (HS-SPME-GC-MS) to analyze volatile compounds and physicochemical properties in four vintages, finding no significant differences in reducing sugar and total acid content, but observing variations in pH and amino acid nitrogen content; a total of 120 compounds were detected—including 9 alcohols, 21 aldehydes, 7 acids, 24 esters, 17 ketones, 7 phenols, 11 alkanes, 3 alkenes, and 21 heterocyclics [5]. Additionally, Chen et al. (2024) optimized the saccharification process by supplementing α-amylase and glucoamylase; results indicated optimal parameters of 2.272% α-amylase addition, liquefaction pH 4.9, temperature 50 °C, and duration 43 min, achieving a reducing sugar content of 5.372 g/100 g [6].

The gut microbiota constitutes a complex ecosystem of bacteria, fungi, viruses, and other microorganisms colonizing the host’s digestive tract—particularly the large intestine—and plays crucial roles in essential physiological processes including nutrient metabolism, immune regulation, and neuromodulation [7]. Dietary components serve as key extrinsic factors modulating the composition and function of intestinal microorganisms, thereby influencing host health through alterations in microbial structure [8,9]. Vinegar, rich in organic acids and bioactive compounds, has been demonstrated to improve intestinal microecological balance by promoting the proliferation of beneficial bacteria [10]. In gut microbiota research, 16S ribosomal RNA (16S rRNA) high-throughput sequencing enables precise microbial identification and classification [11], facilitating assessments of microbial diversity and richness while revealing compositional differences [12]. Short-chain fatty acids (SCFAs), comprising organic acids with one to six carbon atoms—such as acetic, propionic, and butyric acids—are produced through dietary fiber fermentation by gut microorganisms [8]. SCFA concentrations are closely linked to microbial composition and play vital roles in maintaining intestinal homeostasis, immune regulation, and the prevention of metabolic disorders [13,14]. Gas chromatography–mass spectrometry (GC-MS), distinguished by its high resolution, sensitivity, and precise qualitative and quantitative capabilities [15], represents the preferred method for analyzing SCFA profiles [16]. Untargeted metabolomics is a high-throughput analytical approach characterizing metabolic states by quantifying the concentration profiles of all measurable low-molecular-weight metabolites [17,18]; Liquid chromatography and tandem mass spectrometry (LC-MS) metabolomics, offering high sensitivity and throughput, is commonly employed for qualitative and quantitative determinations [19]. Combined with high-resolution mass spectrometry and multivariate analysis, untargeted metabolomics has been successfully applied to evaluate metabolic differences in aged vinegars like Shanxi aged vinegar versus other cereal vinegars, identifying numerous metabolites significantly correlated with aging time [20]. Furthermore, untargeted metabolomics and SCFA detection are complementary: the former comprehensively captures metabolic alterations while the latter quantifies key metabolites with precision; their combination enables comprehensive monitoring of integrated metabolic responses to nutritional interventions [21].

Despite being a regionally distinctive fermented product and National Geographical Indication Protected Product from Yunnan Province, current research on LFAV has largely been confined to process optimization and flavor characterization. Its health-promoting functions—particularly regarding the regulation of gut microbiota and associated metabolites—have not been systematically investigated. Therefore, this study employed 16S rRNA sequencing, GC-MS, and LC-MS to investigate the impact of LFAV intervention at varying doses on murine intestinal microbiota structure, short-chain fatty acids, and metabolic profiles, thereby exploring LFAV’s potential health benefits. The findings will establish a theoretical foundation for its utilization as a functional food ingredient and for the development of functional vinegar-based products.

## 2. Materials and Methods

### 2.1. Reagents and Materials

LFAV (2017 vintage) was sourced from a commercial vinegar manufacturer in Lufeng County, China. Ethyl ether was acquired from Titan (Shanghai, China), while phosphoric acid was sourced from Sinopharm (Shanghai, China). Authenticated SCFA standards including acetic acid, propionic acid, butyric acid, isobutyric acid, isovaleric acid, valeric acid, 4-methylvaleric acid, and caproic acid were procured from Sigma-Aldrich (Shanghai, China). LC-MS grade acetonitrile (ACN) and formic acid were purchased from Fisher Scientific (Loughborough, UK) and TCI (Shanghai, China), respectively. Ammonium formate was obtained from Sigma-Aldrich (Shanghai, China), with ultrapure water generated via a Milli-Q purification system (Millipore, Bedford, MA, USA). The internal standard 2-amino-3-(2-chloro-phenyl)-propionic acid was supplied by Aladdin (Shanghai, China).

### 2.2. Instruments

Refrigerated and high-speed freezing centrifuges were procured from Hunan Xiangyi Experiment Equipment Co., Ltd. (Changsha China). A high-throughput tissue grinder and a tissue grinder were acquired from Zhejiang Meibi Instrument Co., Ltd. and Zhejiang Meibi Experiment Equipment Co., Ltd. (Jiaxing, China). Vortex mixers were supplied by Haimen Kylin-bell Lab Instruments Co., Ltd. (Haimen, China). An ultrasonic cleaner was purchased from Kunshan Shumei Experiment Equipment Co., Ltd. (Kunshan, China), and microporous membrane filters (0.22 µm) were obtained from Tianjin Jinteng Experiment Equipment Co., Ltd. (Tianjin, China). The centrifugal vacuum evaporator was provided by Eppendorf China Ltd. (Shanghai, China). GC analysis was conducted using a Trace 1300 gas chromatograph (Thermo Fisher Scientific, Waltham, MA, USA), while mass spectrometric detection of metabolites was performed on an ISQ 7000 instrument (Thermo Fisher Scientific). The Vanquish liquid chromatograph and Orbitrap Exploris 120 mass spectrometer were obtained from Thermo Fisher Scientific.

### 2.3. Mouse Grouping and Experimental Design

Five-week-old, healthy male Kunming mice were obtained from Henan Sukeibaisi Biological Technology Co., Ltd. (Anyang, China). Prior to experimentation, all mice were acclimated for one week with ad libitum access to food and water. Subsequently, the forty mice were randomly divided into four groups (*n* = 10/group) receiving daily gavage treatments: the control (B) group was administered distilled water, while experimental groups received undiluted LFAV. Gavage doses were adjusted according to daily body weight at the following levels: low-dose (M-L) group 3.3 mL/kg, medium-dose (M-M) group 4.95 mL/kg, and high-dose (M-H) group 6.6 mL/kg (experimental grouping detailed in Table S1). Following 37 consecutive days of LFAV administration, mice were fasted for 12 h post-final gavage. Blood was then collected via orbital puncture, animals were euthanized by cervical dislocation, and intestinal fecal samples were collected for 16S rRNA sequencing, GC-MS, and LC-MS analyses [22].

### 2.4. 16S rRNA Gene Sequencing

Following extraction from fecal samples with the TIANamp Stool DNA Kit, DNA concentration and purity were assessed with a microvolume spectrophotometer, and integrity was confirmed via 0.8% agarose gel electrophoresis. The V3–V4 hypervariable region of the microbial 16S rRNA gene was then amplified by PCR using this DNA as the template. The 25 μL reaction mixture contained 5 μL 5× reaction buffer, 5 μL 5× GC buffer, 2 μL dNTPs (2.5 mM), 1 μL each of forward and reverse primers (10 μM), 2 μL DNA template, 8.75 μL ddH_2_O, and 0.25 μL Q5 High-Fidelity DNA Polymerase. Thermal cycling parameters comprised initial denaturation at 98 °C for 2 min, followed by 25–30 cycles of 98 °C for 15 s, 55 °C for 30 s, and 72 °C for 30 s, with final extension at 72 °C for 5 min. Purified amplicons were subjected to paired-end sequencing on the Illumina MiSeq platform (San Diego, CA, USA) [23]. Raw sequencing data were processed through QIIME2 for bioinformatic analysis, including quality filtering, operational taxonomic unit (OTU) clustering, taxonomic classification, and diversity index calculations.

### 2.5. SCFA Quantification by GC–MS

#### 2.5.1. Sample Preparation

An appropriate quantity of intestinal fecal sample was transferred to a 1.5 mL centrifuge tube. Subsequently, 500 μL of water and 100 mg of glass beads were added. The mixture was homogenized for 1 min, followed by centrifugation at 12,800× *g* (4 °C) for 10 min. From the resulting supernatant, 200 μL was collected and transferred to a new tube. To this aliquot, 100 μL of 15% phosphoric acid was added, followed by 20 μL of internal standard solution (375 μg/mL 4-methylvaleric acid) and 280 μL of ethyl ether. The mixture was again homogenized for 1 min and centrifuged at 12,800× *g* (4 °C) for 10 min. The final supernatant was collected for GC-MS analysis [24].

#### 2.5.2. GC-MS Analysis

SCFA analysis was conducted using a Thermo Trace 1300 gas chromatography system (Thermo Fisher Scientific, USA) equipped with an Agilent HP-INNOWAX capillary column (30 m × 0.25 mm ID × 0.25 μm film thickness). Chromatographic separation was achieved under split injection mode (10:1 ratio) with a 1 μL injection volume. Temperature parameters were maintained as follows: injector at 250 °C, ion source at 300 °C, and transfer line at 250 °C. The oven temperature program initiated at 90 °C, followed by sequential ramps of 10 °C/min to 120 °C, 5 °C/min to 150 °C, and 25 °C/min to 250 °C (held for 2 min), using helium carrier gas at a constant flow rate of 1.0 mL/min. Mass spectrometric detection was conducted on a Thermo ISQ 7000 instrument (Thermo Fisher Scientific) operated in electron ionization (EI) mode with selected ion monitoring (SIM), employing 70 eV ionization energy [25].

### 2.6. Untargeted Metabolomics

#### 2.6.1. Metabolite Extraction

Precisely weighed intestinal fecal samples were transferred to 2 mL centrifuge tubes. Exactly 1000 μL of tissue extraction solution [75% (9:1 methanol: chloroform): 25% H_2_O] was added, followed by steel beads. Tubes were placed in a tissue homogenizer and homogenized at 50 Hz for 60 s, with this process repeated twice. Subsequently, samples underwent room temperature ultrasonication for 30 min followed by 30 min incubation on ice. Centrifugation was performed at 12,800× *g* (4 °C) for 10 min, after which the entire supernatant was transferred to new tubes, concentrated, and dried. The residue was reconstituted in 200 μL of 50% acetonitrile solution containing 4 ppm 2-chloro-L-phenylalanine. Finally, the filtered solution was transferred to vials for LC-MS analysis [26].

#### 2.6.2. Untargeted Metabolomics Measurement

Metabolites were extracted using 75% methanol-chloroform. Chromatographic separation was achieved on a Thermo Vanquish UHPLC system (Thermo Fisher Scientific) equipped with an ACQUITY UPLC^®^ HSS T3 column (2.1 × 100 mm, 1.8 µm; Waters, Milford, MA, USA) maintained at 40 °C with a flow rate of 0.3 mL/min and injection volume of 2 μL. For positive ion mode analysis, mobile phases consisted of 0.1% formic acid in acetonitrile (B1) and 0.1% formic acid in water (A1) using the following gradient: 0–1 min (8% B1), 1–8 min (8–98% B1), 8–10 min (98% B1), 10–10.1 min (98–8% B1), 10.1–12 min (8% B1). Negative ion mode analysis employed acetonitrile (B2) and 5 mM ammonium formate in water (A2) with identical gradient parameters [27]. Mass spectrometric detection was performed using a Thermo Orbitrap Exploris 120 instrument (Thermo Fisher Scientific) equipped with an electrospray ionization (ESI) source. Data acquisition was conducted in both positive and negative ion modes with spray voltages set at 3.50 kV and −2.50 kV, respectively. Instrument parameters were optimized as follows: sheath gas flow maintained at 40 arbitrary units (arb), auxiliary gas at 10 arb, and capillary temperature stabilized at 325 °C. Full scan MS1 spectra were acquired at a resolution of 60,000 across the m/z range 100–1000, followed by data-dependent MS2 acquisition through higher-energy collisional dissociation (HCD) at 30% normalized collision energy. MS2 spectra were collected at 15,000 resolution, with the top four most intense precursor ions selected for fragmentation per cycle. Dynamic exclusion was implemented to prevent redundant MS/MS fragmentation of previously analyzed ions [28].

### 2.7. Statistical Analysis

The study data are expressed as means ± standard deviation. Statistical comparisons were performed using one-way ANOVA in SPSS Statistics 21 software, followed by post hoc tests where applicable. Statistically significant differences (*p* < 0.05) between groups are indicated by different lowercase letters. Pearson correlation analyses and graphical visualizations were generated with Origin 2019, while correlation heatmaps were constructed using TBtools software (version 2.330). Bioinformatics analysis and visualization were conducted in QIIME2 (2019 version) and R software (version 4.0.3). Functional pathway enrichment analysis of screened differential metabolites was performed using the MetaboAnalyst software package. Pathways identified through enrichment were subsequently mapped and visualized with the KEGG Mapper tool to illustrate differential metabolites within pathway contexts.

## 3. Results and Discussion

### 3.1. Effects of LFAV on Taxonomic Units of Intestinal Microbiota in Mice

A Venn diagram clearly illustrated the distribution of shared OTUs across the four experimental groups (Figure S1). The analysis identified 269 OTUs common to all groups, while unique OTU counts were 400 (M-L group), 312 (M-M group), 291 (M-H group), and 274 (B group). Notably, the M-L group exhibited the highest proportion of unique OTUs (19.98%), which significantly exceeded those of the M-M group (14.69%) and B group (13.69%). This pattern suggests an inverse dose-dependent effect of LFAV on species richness in murine gut microbiota, wherein reduced dosing correlated with increased OTU counts and enhanced microbial richness. Intestinal pH, through its influence on microbial growth conditions, critically regulates gut microbiota composition and metabolic activities [29]. The elevated unique OTU count in the M-L group may be attributed to pH modulation by organic acids (e.g., acetic acid) promoting microbial proliferation, potentially augmented by polyphenols that may maintain microbial homeostasis through antioxidative and anti-inflammatory pathways [30]. In contrast, the high acetic acid concentration in the M-H group may lower pH beyond optimal levels, thereby suppressing microbial proliferation and reducing microbiota richness.

### 3.2. Effects of LFAV on Alpha and Beta Diversity of Intestinal Microbiota

**Figure 1:**
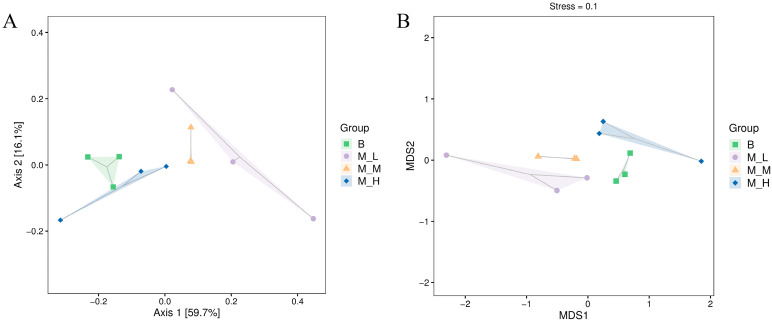
The effect of Lufeng aromatic vinegar on beta diversity indices of intestinal microbiota in mice: (**A**) 2D ordination plot of PCoA; (**B**) NMDS plot.

Alpha diversity analysis (Table S2) revealed a gradient in species richness via the Chao1 index, with the M-L group (496.63 ± 42.14) demonstrating significantly higher values than the M-M (480.52 ± 59.36), M-H (438.97 ± 90.53), and B (362.21 ± 129.54) groups. Coverage indices (mean 1.00) confirmed sufficient sequencing depth to capture most intestinal bacteria with high reliability. For diversity indices, the M-L group showed increased Shannon (6.68 ± 0.32) and Simpson (0.97 ± 0.01) indices relative to the B group (3.25% and 2.11% higher, respectively), exceeding values in other treatment groups. Although ANOVA indicated no statistically significant between-group differences (*p* > 0.05), an inverse dose-dependent pattern was observed: increasing administration dose correlated with decreasing OTU counts (400 → 312 → 291), Shannon index (6.68 → 5.38), and Simpson index (0.97 → 0.92). Beta diversity analysis (Figure 1) demonstrated that LFAV dosage significantly influenced gut microbiota structure. In principal coordinates analysis (PCoA) (Figure 1A), the first principal coordinate (59.7% explanation) distinctly separated the M-L group from others, showing positive displacement along PCo1 inversely correlated with dosage. Partial overlap between the M-H and B groups suggested structural convergence. Non-metric multidimensional scaling (NMDS) analysis (Figure 1B) further confirmed the dose–response relationship: the M-L group exhibited greater separation along MDS1 compared to M-M and M-H groups, while M-H group clustered near the B group. The reduced structural divergence at higher doses may involve compensatory microbial adaptation mechanisms [31]. These findings align with prior vinegar research: Xia et al. (2022) documented vinegar extract (VE) modulating Bacteroidetes, Lactobacillus, and Bifidobacterium abundance in T2DM mice [32], while Geng et al. (2021) reported concentrated vinegar solids (CVS) enriching beneficial *Akkermansia* and *Lactobacillus* while suppressing undesirable *Desulfovibrio* and *Clostridium* sensu stricto 1 in mice [33].

### 3.3. Effects of LFAV on Gut Microbiota Composition and Taxonomic Shifts

**Figure 2:**
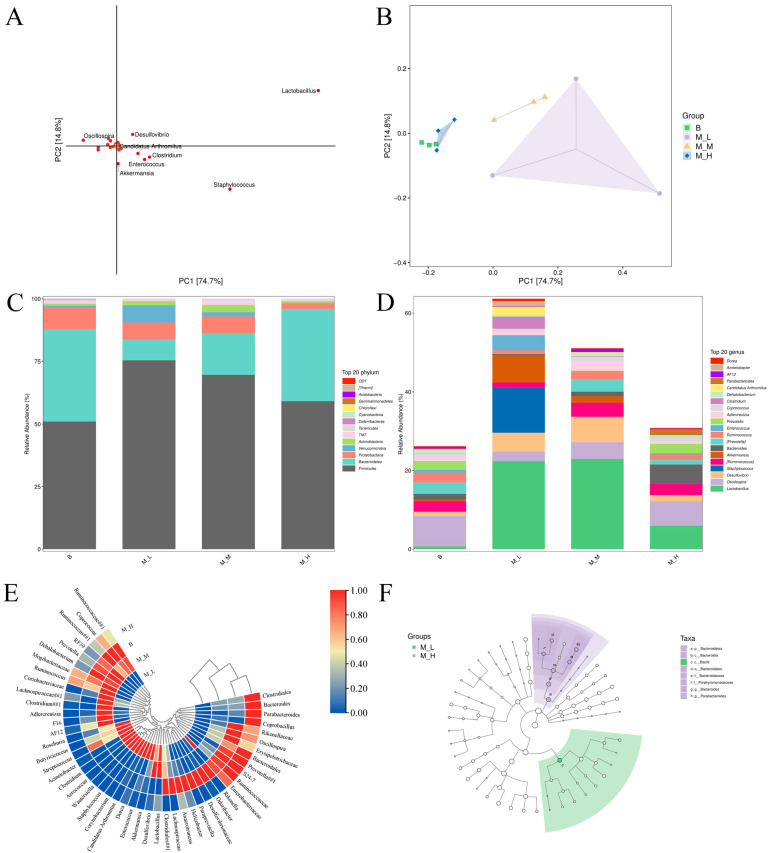
The effects of Lufeng aromatic vinegar on gut microbiota composition and taxonomic shifts: (**A**) PCA loading plot, (**B**) PCA score plot, (**C**) phylum-level taxonomic bar chart, (**D**) genus-level taxonomic bar chart, (**E**) clustered heatmap, (**F**) phylogenetic tree.

Principal component analysis (PCA) of spatial variation patterns (Figure 2A,B) revealed differential impacts of LFAV dosages on murine gut microbiota composition. High cumulative variance explanation (PC1: 74.7%; PC2: 14.8%) indicated robust model resolution and effective discrimination between samples [34]. The PCA score plot (Figure 2B) demonstrated close clustering between the M-H and B groups, indicating that high-dose intervention yielded microbial profiles approaching baseline conditions, consistent with preceding alpha and beta diversity findings. Conversely, the M-L and M-M groups (particularly M-L group) exhibited significant displacement along the PC1 axis and greater dispersion relative to the B group, suggesting more pronounced structural alterations at lower doses. Loading plot analysis (Figure 2A) identified *Lactobacillus* as key discriminating taxa, implying LFAV may modulate gut microbiota structure through selective proliferation of beneficial bacteria. Although *Lactobacillus* plays essential roles in traditional fermentation, its growth is frequently suppressed by acetic acid bacteria under conditions of elevated ethanol and organic acid concentrations [35]. The comparatively lower organic acid levels in the M-L group versus higher-dose interventions in the M-H group likely created favorable conditions for lactobacilli proliferation.

Analysis of phyla with mean relative abundance exceeding 0.1% (Figure 2C) revealed the B group gut microbiota was dominated by Firmicutes (51.0%) and Bacteroidetes (36.9%), collectively constituting 87.9% of the baseline community. A structural shift was observed in the M-L group, where Firmicutes abundance significantly increased to 75.4% while Bacteroidetes decreased to 8.2%, establishing Firmicutes as the core phylum. The M-M group displayed transitional characteristics with Bacteroidetes rising to 16.4% and Firmicutes to 69.7%. Verrucomicrobia exhibited substantial proliferation in the M-L group, reaching 6.9% (a 138-fold increase over B group’s 0.05%). The M-H group showed partial compositional regression, with Firmicutes (59.1%) and Bacteroidetes (36.8%) approximating B group levels, while Proteobacteria abundance decreased significantly to 2.2% (75% reduction from B group’s 8.8%). A visual comparison of the color distribution in the bar charts across the four groups reveals a notable expansion of the gray segment (Firmicutes) in the M-L group, accompanied by a substantial contraction of the green segment (Bacteroidetes). This distinct visual pattern provides clear evidence that LFAV exerts a significant regulatory effect on the core bacterial phyla, which varies with its dosage level. The proliferation of Firmicutes in the M-L and M-M groups may be attributed to pH modulation by optimal concentrations of acetic acid and other organic acids, which can suppress pathogenic bacteria [36] and influence microbial ecology.

Dominant genera were defined as the top 20 most abundant taxa based on mean relative abundance (Figure 2D). In the M-L group, *Lactobacillus* (22.39%), *Staphylococcus* (11.39%), and *Akkermansia* (6.93%) demonstrated significant proliferation, whereas unclassified S24-7 (22.29% in B group) decreased to 6.62%. The M-M group maintained high *Lactobacillus* abundance (22.94%), with increased proportions of unclassified S24-7 (9.44%) and *Desulfovibrio* (6.45%) compared to the M-L group. The M-H group exhibited compositional reversion toward the B group patterns, dominated by *Clostridiales* (23.70%) and S24-7 (22.44%), though Lactobacillus abundance (5.96%) remained substantially higher than in the B group (0.32%). These findings indicate that low-dose intervention promotes proliferation of beneficial taxa including Lactobacillus and *Akkermansia*. *Lactobacillus* enhances intestinal barrier function, modulates microbial balance, regulates innate immunity, and inhibits pathogen colonization [37]. *Akkermansia*, a mucin-degrading Gram-negative obligate anaerobe inhabiting the intestinal mucus layer, produces propionate, stimulates goblet cell differentiation, maintains mucosal integrity, and promotes host-microbiota equilibrium, establishing its potential as a probiotic [38,39]. This aligns with Zhai et al.’s finding that lactulose promotes *Akkermansia* proliferation to improve host health [40].

The cluster heatmap constructed from genus-level abundance data (Figure 2E) provided visual evidence of microbial compositional differences between experimental groups. A clear differentiation was observed: the M-L and M-M groups formed a single cluster branch due to highly similar microbial profiles, while the M-H and B groups formed distinct clusters. Analysis of dominant genera revealed partial overlap in high-abundance taxa (red regions) between the M-H and B groups, indicating shared microbial characteristics. Limited overlap was detected between the M-M and B groups, whereas no shared high-abundance genera were observed between the M-L and B groups. This spatial separation pattern aligned with previously described PCA results, further demonstrating that low-dose LFAV intervention effectively modulates gut microbiota structure.

To further elucidate the relationship between LFAV dosage and gut microbiota composition, LEfSe analysis was performed (Figure 2F). The radial cladogram illustrated microbial taxonomy from phylum to genus levels (innermost to outermost rings, respectively), with unmarked nodes indicating taxa showing no significant differences (*p* > 0.05) between the M-L and M-H groups, while annotated nodes denoted statistically differential taxa (*p* ≤ 0.05) [41]. Biomarker selection employed the LEfSe algorithm with thresholds set at *p* < 0.05 (Kruskal–Wallis test) and LDA score > 2 (Figure S2). Linear discriminant analysis (LDA) integrated statistical significance with effect size quantification, where higher scores indicated greater explanatory power for intergroup differences [42]. Significant structural variations between the M-L and M-H groups were primarily driven by: Bacteroidetes (phylum), Bacteroidaceae and unclassified Porphyromonadaceae (family), *Bacteroides* and *Parabacteroides* (genus), and Bacteroidia and Bacilli (class). These taxa exhibited significant relative abundance differences (*p* < 0.05) with LDA scores exceeding 4. Complementary data (Figure 2D) demonstrated that Bacteroides abundance increased proportionally with dosage (2.75% → 11.58% → 48.58%), whereas *Lactobacillus* and *Akkermansia* showed inverse correlations with dosage, confirming differential regulatory effects of LFAV concentrations on gut microbiota.

Collectively, these findings demonstrate that LFAV intervention at low and medium doses (M-L and M-M groups) significantly altered murine gut microbiota structure, promoting probiotic proliferation (notably *Lactobacillus*) and enhancing microbial richness. Zhang et al. (2023) documented that *Lactobacillus* plantarum L168 and its metabolite indole-3-lactic acid ameliorate intestinal inflammation, tumor growth, and dysbiosis [43]. The significant increase in probiotics such as *Lactobacillus* following the intervention with LFAV suggests that it may have potential positive effect on promoting host intestinal health. This highlights the value of LFAV as a potential functional food ingredient. However, the relatively modest regulatory effect of the M-H group on gut microbiota may be attributed to its strong acidity, which could exceed the tolerance threshold of certain bacterial species [44]. As the M-H group (6.6 mL/kg) represents the highest dose administered in this study, a non-linear dose–response relationship has been observed under this condition, where the regulatory effect of the M-H group was significantly lower than those of the M-L and M-M groups. In addition, the OUT composition of the M-H group was found to be similar to that of the B group. Therefore, it is suggested that adverse effects may also occur with LFAV intervention at even higher doses. It is worth noting that in a study by Xia et al. (2024), mice treated with 2 mL/kg of Shanxi aged vinegar exhibited significant modulation of intestinal flora, including an increase in *Akkermansia* and other beneficial bacteria [22]. Similarly, in research by Cao et al. (2024), intervention with 2 mL/kg of Cornus officinalis vinegar in mice was found to significantly increase the abundance of beneficial bacteria such as *Lactobacillus* and *Bifidobacterium*, suppress certain harmful bacteria such as Bacteroides, and restore gut microbiota diversity [45]. These findings are consistent with the results obtained in the present study. Therefore, it is suggested that medium and low doses of LFAV may possess greater potential as raw or auxiliary materials for functional foods.

### 3.4. Effects of LFAV on SCFA Profiles in Mice

SCFAs, recognized as major gut microbiota metabolites, contribute to intestinal health by reducing luminal pH to inhibit pathogens and maintain mucosal barrier integrity [46]. LFAV intervention significantly altered cecal SCFA profiles (Table 1). Acetate, butyrate, and valerate concentrations were significantly elevated (*p* < 0.05) in all treatment groups versus the B group, with acetate peaking in M-H group (3248.97 ± 146.79 μg/g). This supports the hypothesis that elevated acetate concentrations may suppress microbial proliferation, consistent with prior Venn and alpha/beta diversity analyses. Notably, butyrate reached its highest concentration in M-L group (1786.51 ± 148.50 μg/g). As butyrate enhances beneficial bacterial activity and metabolic function [47], its elevation may explain the observed proliferation of Lactobacillus in M-L and M-M groups (Figure 2D). The M-M group exhibited distinct SCFA characteristics: significantly higher valerate (86.98 ± 1.63 μg/g), isovalerate (35.95 ± 1.18 μg/g), propionate (642.74 ± 32.72 μg/g), and isobutyrate (66.83 ± 4.31 μg/g) compared to other groups. This pattern may be attributed to vinegar-induced alterations in microbial fermentation substrates [48]. Propionate production via succinate or acrylate pathways involves *Clostridium* and *Lactobacillus* species [49], while *Clostridium* primarily generates isobutyrate and isovalerate [50]. Elevated abundances of these genera in M-L and M-M groups (Figure 2D) correspond to increased production of these SCFAs. Collectively, LFAV dosage differentially modulates SCFA composition, with the M-M group exhibiting the most significant regulatory effect, followed by the M-L group.

### 3.5. Untargeted Metabolomics Analysis

#### 3.5.1. Differential Metabolite Profiling

**Figure 3:**
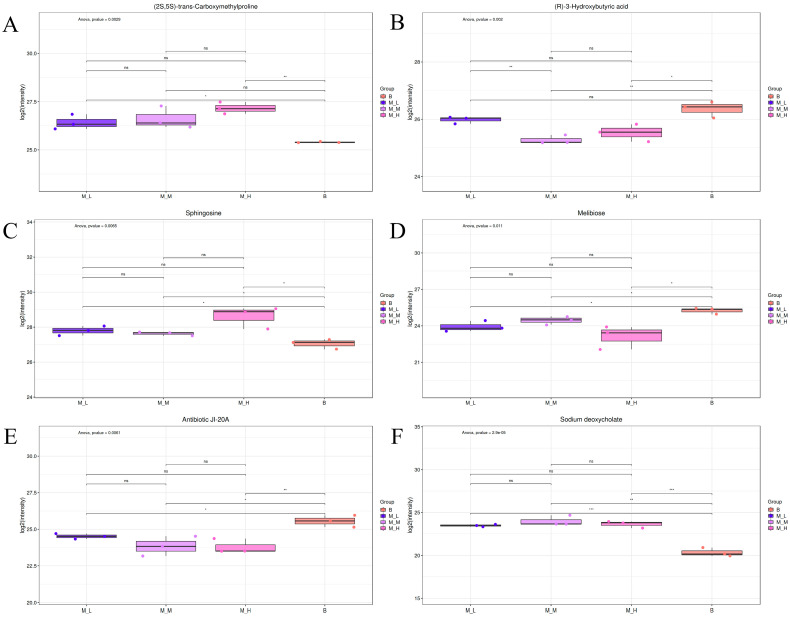
Boxplot of the top 6 VP value metabolites in a non-targeted metabolomics study: (**A**) (2S,5S)-transCarboxymethylproline, (**B**) (R)-3-Hydroxybutyric acid, (**C**) Sphingosine, (**D**) Melibiose, (**E**) Antibiotic J1-20A, and (**F**) Sodium deoxycholate. * indicates *p* < 0.05, ** indicates *p* < 0.01, *** indicates *p* < 0.001, and ns indicates *p* > 0.05.

Untargeted metabolomics was employed to investigate the effects of varying LFAV doses on murine cecal metabolites. Metabolite identification was achieved through fragment ion matching analysis against multi-source spectral databases (HMDB, MassBank, LipidMaps, mzCloud, KEGG, and Nuomimeta’s in-house standard database), resulting in the annotation of 520 metabolites. Based on primary differential analysis criteria (VIP ≥ 1, *p* < 0.05), 60 statistically significant secondary differential metabolites were identified (Table S3). These metabolites comprised: amino acid derivatives (7), fatty acids (6), carboxylic acids (5), saccharides (4), indole derivatives (3), amino acids (2), purine bases (2), alkaloids (2), peptides (2), vitamins (2), amides (2), heterocyclic compounds (2), and esters (2), with 19 metabolites representing unique chemical classes. The variable importance in projection (VIP) metric, which evaluates feature importance by considering both X and Y variable correlations [51], was utilized for variable selection; metabolites with VIP scores exceeding 1 were considered biologically significant. Consequently, the six highest-ranking VIP metabolites were selected for box plot visualization (Figure 3).

Box plots (Figure 3A–F) visualized the top six VIP-ranked differential metabolites, with group assignments on the *x*-axis and quantitative values on the *y*-axis; statistical significance was denoted by asterisks. The highest-VIP metabolite, (2S,5S)-trans-Carboxymethylproline (Figure 3A), demonstrated elevated concentrations in all intervention groups versus the B group, with significant (M-L group vs. B group: *p* < 0.05) and highly significant (M-H group vs. B group: *p* < 0.01) differences, which showed an increasing trend with higher doses. This compound, biosynthesized via carboxymethylproline synthase-mediated condensation of L-glutamic semialdehyde and malonyl-CoA [52], was identified by Du et al. [53] as a potential protective gut metabolite through logistic regression modeling. Its elevation following LFAV intervention supports the intestinal health-promoting effects of vinegar. Metabolites ranked 2nd, 4th, and 5th by VIP score [(R)-3-Hydroxybutyric acid (Figure 3B), Melibiose (Figure 3D), Antibiotic JI-20A (Figure 3E)] exhibited significantly higher concentrations in the B group compared to intervention groups (*p* < 0.05). (R)-3-Hydroxybutyric acid participates in ketone body metabolism [54] and has been associated with schizophrenia [55]. The 3rd and 6th VIP-ranked metabolites [Sphingosine (Figure 3C), Sodium deoxycholate (Figure 3F)] showed increased concentrations in intervention groups versus the B group. Sphingosine, a biologically active sphingolipid, inhibits protein kinase C and blocks staphylococcal enterotoxin B-induced effects [56]. Sodium deoxycholate, an anionic bile salt, reduced acetylcholine effects in mesenteric vascular beds by 97.2% in experimental treatments [57].

#### 3.5.2. Clustering and Z-Score Analysis

**Figure 4:**
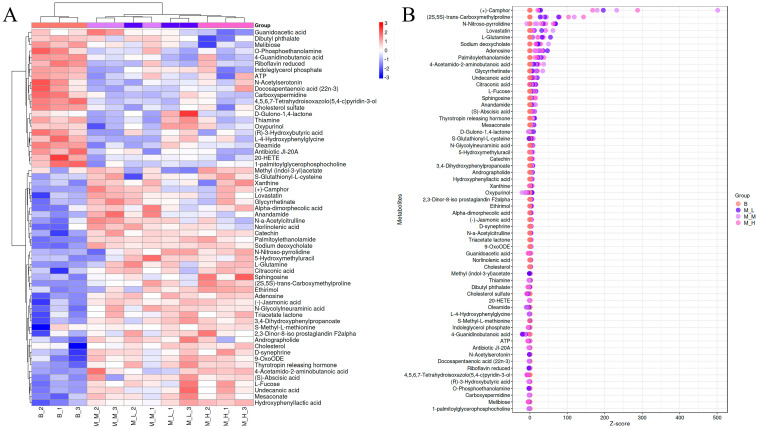
Cluster analysis and Z-score analysis were performed on untargeted metabolites: (**A**) clustering heatmap; (**B**) Z-score plot.

To further analyze the impact of LFAV intervention on murine cecal metabolites, cluster heatmap (Figure 4A) and Z-score normalization analyses (Figure 4B) were performed on the 60 secondary differential metabolites. The heatmap employed a color gradient from blue (low abundance) to red (high abundance), with sample clustering demonstrating tight grouping of biological replicates within experimental groups, indicating strong reproducibility. The B group exhibited characteristically elevated levels of metabolites including O-Phosphoethanolamine, N-Acetylserotonin, Docosapentaenoic acid (22n-3), Carboxyspermidine, (R)-3-Hydroxybutyric acid, Melibiose, and Antibiotic JI-20A. The expression patterns of (R)-3-Hydroxybutyric acid, Melibiose, and Antibiotic JI-20A in the heatmap aligned with box plot results (Figure 3B,D,E), consistently showing significantly higher abundance in the B group versus intervention groups. Furthermore, high-abundance metabolites in the M-L, M-M, and M-H groups showed substantial overlap while distinctly differing from the B group profile, suggesting these metabolites may originate from LFAV’s bioactive components or represent intervention-specific biosynthetic products. Z-score normalization quantified relative metabolite abundance differences, facilitating visual comparison of overall metabolic trends between experimental and control groups. As shown in Figure 4B, the *x*-axis represents Z-scores and the *y*-axis metabolite names, with rightward displacement indicating higher relative abundance. Distinct distribution patterns were observed between the B group and intervention groups, with pronounced differences in metabolites including (+)-Camphor, (2S,5S)-trans-Carboxymethylproline, N-Nitroso-pyrrolidine, Lovastatin, Sodium deoxycholate, Adenosine, Palmitoylethanolamide, 4-Acetamido-2-aminobutanoic acid, and Glycyrrhetinate. The Z-score distributions of key differential metabolites (2S,5S)-trans-Carboxymethylproline (VIP rank 1) and Sodium deoxycholate (VIP rank 6) corresponded closely with their respective box plots (Figure 3A,F). Collectively, these analyses demonstrate that LFAV intervention significantly modulates cecal metabolite profiles, with marked compositional differences between control and treatment groups.

#### 3.5.3. KEGG Pathway Enrichment Analysis

**Figure 5:**
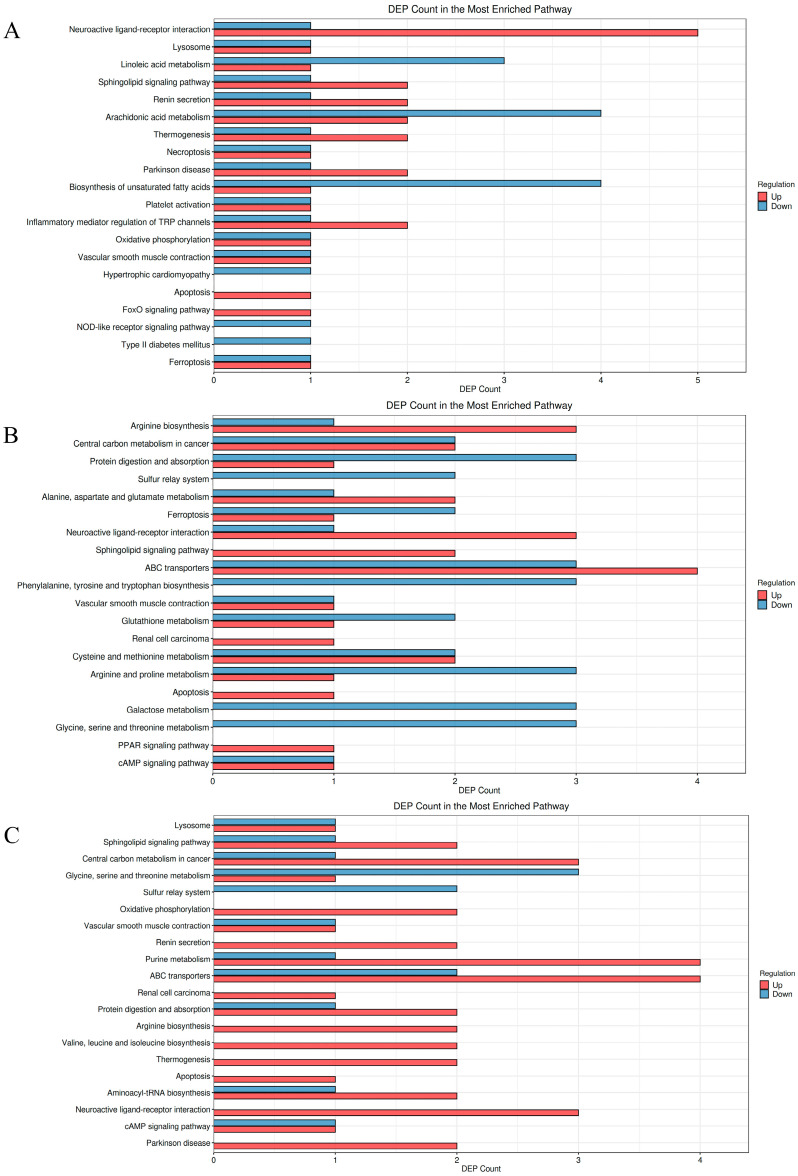
Statistical plot of differential enrichment counts: (**A**) M-L group vs. B group; (**B**) M-M group vs. B group; (**C**) M-H group vs. B group.

Kyoto Encyclopedia of Genes and Genomes (KEGG) pathway enrichment analysis, a widely utilized bioinformatics approach [58], was employed to investigate metabolic alterations following LFAV intervention. Differential metabolites were mapped to KEGG pathways, with results visualized in Figure 5 where the *x*-axis represents metabolite counts and the *y*-axis denotes metabolic pathways. Red and blue bars indicate upregulated and downregulated pathways, respectively. Figure 5A (M-L group vs. B group), 5B (M-M group vs. B group), and 5C (M-H group vs. B group) collectively revealed significant pathway alterations across intervention groups. The M-L group versus the B group comparison (Figure 5A) exhibited notable upregulation in neuroactive ligand–receptor interactions, sphingolipid signaling, renin secretion, Parkinson’s disease, and TRP channel inflammatory mediation pathways. Neuroactive ligand–receptor interactions demonstrated the most pronounced upregulation—a pathway involving ligand–receptor binding that triggers signaling cascades regulating neuronal communication, cellular functions, and metabolic homeostasis, with established roles in gonadal steroidogenesis [59] and predicted significance in diabetes regulation [60].

When comparing the M-M group to the B group (Figure 5B), upregulated pathways included arginine biosynthesis; alanine, aspartate, and glutamate metabolism; neuroactive ligand–receptor interactions; ABC transporters; and sphingolipid signaling pathways, with arginine biosynthesis and neuroactive ligand–receptor interactions demonstrating particularly significant upregulation. Conversely, downregulated pathways encompassed protein digestion and absorption, sulfur relay systems, ferroptosis, glutathione metabolism, arginine and proline metabolism, galactose metabolism, and glycine, serine, and threonine metabolism. The arginine biosynthesis pathway represents a fundamental biochemical route for arginine production, catalyzing the conversion of carbon sources (e.g., glutamate or glutamine) into arginine while integrating nitrogen and energy metabolism. Metabolites within this pathway (e.g., ornithine, glutamine, arginine) have been implicated in oxidative stress regulation, immune responses, and inflammation suppression [61]. Galactose metabolism facilitates the biochemical conversion of galactose into utilizable energy or structural molecules, with impaired function of the Leloir pathway known to cause galactosemias [62].

Comparative analysis of the M-H group versus the B-group (Figure 5C) revealed upregulated pathways including sphingolipid signaling, central carbon metabolism in cancer, oxidative phosphorylation, renin secretion, purine metabolism, ABC transporters, protein digestion and absorption, arginine biosynthesis, valine, leucine, isoleucine biosynthesis, apoptosis, aminoacyl-tRNA biosynthesis, neuroactive ligand–receptor interactions, and Parkinson’s disease. Among these, purine metabolism and neuroactive ligand–receptor interactions demonstrated particularly significant upregulation. Conversely, glycine, serine, and threonine metabolism and sulfur relay systems were downregulated. Purine metabolism represents the core biochemical process for purine nucleotide synthesis and degradation, with uric acid serving as its terminal metabolite that contributes to salivary antioxidant properties [63].

Collectively, neuroactive ligand–receptor interactions were consistently observed to be significantly upregulated across the M-L, M-M, M-H groups versus the B group in comparative analyses, establishing this pathway as a core upregulated metabolic pathway under LFAV intervention. This persistent upregulation may be attributed to acetate serving as an acetyl-CoA precursor that directly fuels the tricarboxylic acid (TCA) cycle and mitochondrial ATP production, thereby providing energetic support for synaptic transmission. Additionally, acetate metabolites potentially activate potassium channels (e.g., KCNQ2/3) to maintain neuronal resting membrane potential and prevent synaptic fatigue from overexcitation [64]. Vinegar intervention collectively induced upregulation across multiple metabolic pathways, with increasing dosage correlating with progressively enhanced upregulation effects. The M-H group exhibited the most pronounced metabolic pathway upregulation, demonstrating a significant positive dose-dependent response in metabolic modulation.

### 3.6. Correlation Analysis

**Figure 6:**
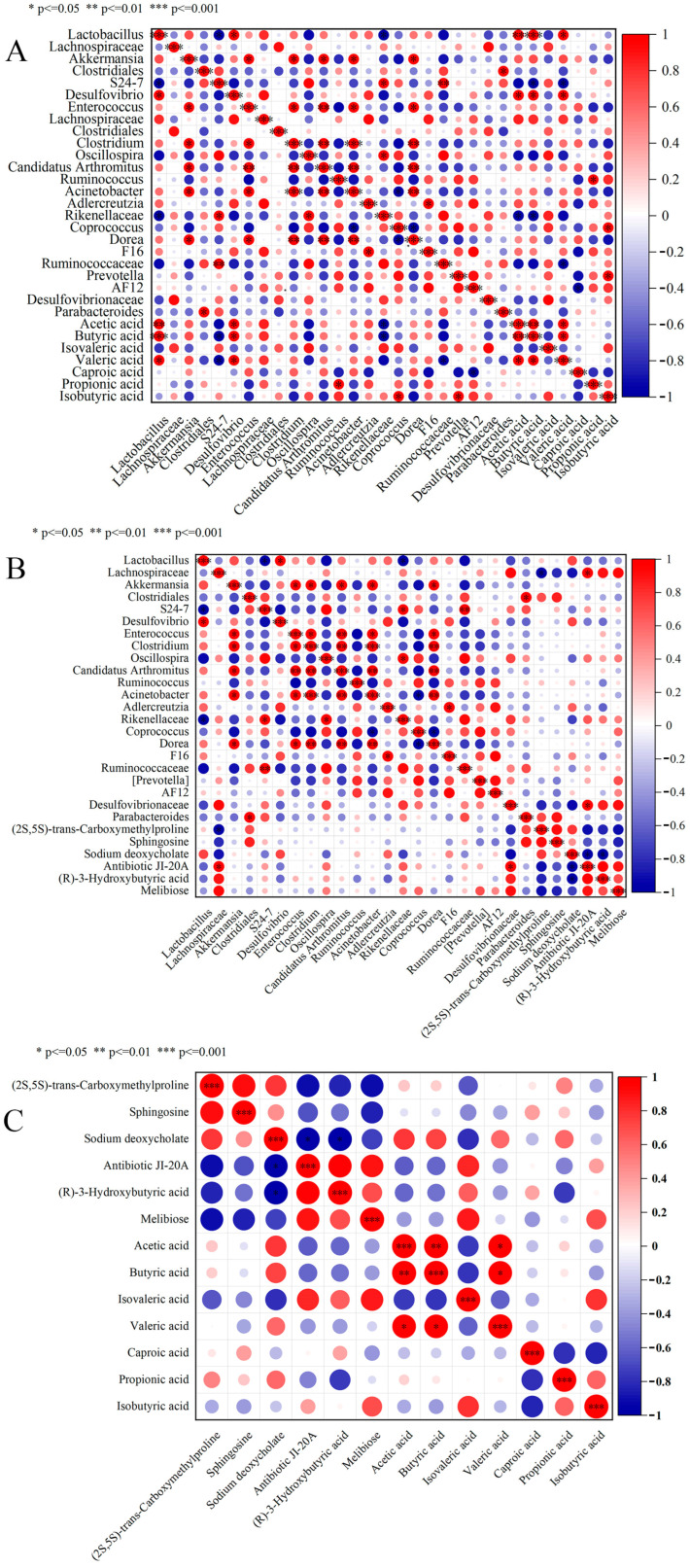
Pearson correlation analysis plot: (**A**) correlation analysis between gut microbiota at the genus level and SCFAs; (**B**) correlation analysis between gut microbiota at the genus level and untargeted metabolites; (**C**) correlation analysis between SCFAs and untargeted metabolites. Red indicates positive correlation, blue indicates negative correlation, * indicates *p* < 0.05, ** indicates *p* < 0.01, *** indicates *p* < 0.001.

Correlation analysis between dominant microbial genera and SCFAs was performed (Figure 6A), with red indicating positive correlations, blue negative correlations, and color intensity proportional to the absolute correlation coefficient. Significant positive correlations were observed between acetate, butyrate, valerate and *Lactobacillus*, *Desulfovibrio*, and *Adlercreutzia* (*p* < 0.05 for *Lactobacillus* and *Desulfovibrio*). The acetate and butyrate correlations with *Lactobacillus* may stem from its glycolytic metabolic advantage: lactobacilli metabolize dietary carbohydrates to lactate, which commensal bacteria (e.g., *Desulfovibrio*) subsequently convert to acetate (via acetyl-CoA pathway) or butyrate (via butyryl-CoA: acetate CoA-transferase pathway) [65]. Figure 6B presents correlations between the top six VIP-ranked metabolites and dominant genera, revealing positive associations between *Desulfovibrionaceae*, *Lachnospiraceae* and Antibiotic JI-20A, (R)-3-Hydroxybutyric acid, and Melibiose, with statistically significant correlations between *Desulfovibrionaceae* and Antibiotic JI-20A (*p* < 0.05). Figure 6C illustrates correlations between the top VIP metabolites and SCFAs, showing non-significant positive correlations (*p* > 0.05) between isovaleric acid and Antibiotic JI-20A, (R)-3-Hydroxybutyric acid, and Melibiose, while negative correlations (*p* > 0.05) were observed with (2S,5S)-trans-Carboxymethylproline and Sodium deoxycholate.

Integrated analysis of Figure 2D and Table 1 revealed significantly increased relative abundance of *Lactobacillus* in the M-L and M-M groups, concurrent with peak butyrate concentrations in these groups. This provides direct evidence supporting the theoretical framework that *Lactobacillus* proliferation promotes butyrate production, aligning with Wang et al.’s (2022) findings [66]. Butyrate, a four-carbon short-chain fatty acid naturally produced through microbial fermentation of dietary fiber, exerts multiple physiological functions including maintenance of intestinal barrier integrity, modulation of microbial balance (pathogen suppression and probiotic promotion), and anticancer effects through apoptosis induction and histone deacetylase inhibition [67]. The concurrent elevation of Lactobacillus abundance and butyrate levels in the M-L and M-M groups demonstrates that LFAV intervention potentially functions through bioactive components that stimulate probiotic proliferation, consequently enhancing beneficial metabolite production. This mechanistic interpretation is consistent with Gao et al.’s (2022) research [68]. These findings not only elucidate the microbiota-modulating mechanisms of LFAV but also highlight its potential as a functional food ingredient. Future research should focus on identifying the specific determinant components within LFAV responsible for this regulatory effect.

## 4. Conclusions

This study systematically evaluated the regulatory effects of LFAV on murine gut microbiota composition, SCFAs, and cecal metabolites. High-throughput 16S rRNA sequencing revealed that LFAV intervention significantly modulated gut microbiota diversity, with the M-L group exhibiting 19.98% unique OTUs. The Chao1 index (496.63 ± 42.14) and Shannon index (6.68 ± 0.32) increased by 37.11% and 3.25%, respectively, compared to the B group, indicating enhanced microbial richness. Taxonomic composition analysis demonstrated that Firmicutes dominated at the phylum level in the M-L group (75.4%), while Bacteroidetes decreased to 8.2%. At the genus level, significant proliferation of *Lactobacillus* (22.39%) and *Akkermansia* (6.93%) was observed. GC-MS analysis detected peak butyric acid concentrations in the M-L group. Untargeted metabolomics identified 520 metabolites, with 60 identified as significant differential metabolites. Cluster heatmap and Z-score analyses confirmed substantial metabolic alterations following LFAV intervention. KEGG pathway enrichment analysis revealed that metabolic pathways were increasingly upregulated with higher doses, with the M-H group showing a widespread upregulation. Neuroactive ligand–receptor interactions were consistently upregulated across all dosage groups, indicating this pathway as a core upregulated target. Pearson correlation analysis demonstrated strong positive correlations (*p* < 0.01) between *Lactobacillus* abundance and acetate and butyrate concentrations. The concurrent elevation of Lactobacillus proliferation and butyrate levels in the M-L and M-M groups further substantiates that LFAV intervention promotes beneficial bacterial growth, which may enhance intestinal health. Collectively, these findings demonstrate LFAV’s significant capacity to modify gut microbiota structure and metabolic profiles, providing a scientific foundation for developing functional vinegar products and highlighting its potential as a functional food ingredient.

## 5. Patents

**Patents:** No patents were obtained in connection with this research.

## Figures and Tables

**Table 1:** Determination of short-chain fatty acids in intestinal tract of mice by LFAV.

Group	Acetic Acid (μg/g)	Butyric Acid (μg/g)	Isovaleric Acid (μg/g)	Valeric Acid (μg/g)	Caproic Acid (μg/g)	Propionic Acid (μg/g)	Isobutyric Acid (μg/g)
B	2230.16 ± 159.22 ^d^	1044.04 ± 22.53 ^b^	19.79 ± 8.30 ^b^	78.43 ± 2.51 ^c^	1.14 ± 0.28 ^ab^	558.69 ± 43.03 ^ab^	44.90 ± 0.68 ^b^
M-L	2561.23 ± 251.09 ^b^	1786.51 ± 148.50 ^a^	30.95 ± 4.68 ^a^	84.59 ± 2.16 ^ab^	1.54 ± 0.43 ^a^	476.11 ± 93.66 ^b^	36.19 ± 4.56 ^c^
M-M	3118.86 ± 83.93 ^a^	1759.54 ± 61.66 ^a^	35.95 ± 1.18 ^a^	86.98 ± 1.63 ^a^	0.91 ± 0.29 ^b^	642.74 ± 32.72 ^a^	66.83 ± 4.31 ^a^
M-H	3248.97 ± 146.79 ^a^	1223.56 ± 102.29 ^b^	28.87 ± 0.49 ^ab^	80.99 ± 1.11 ^bc^	1.01 ± 0.17 ^ab^	486.02 ± 84.93 ^b^	40.39 ± 2.62 ^bc^

Note: Different lowercase letters in the same row indicate significant difference (*p* < 0.05), while the same lowercase letters indicate insignificant difference (*p* > 0.05).

## Data Availability

The data presented in this study are available on request from the corresponding author.

## Supplementary Materials

The following supporting information can be downloaded at: https://www.mdpi.com/article/10.3390/foods14213747/s1. Figure S1. Venn diagram of Lufeng aromatic vinegar on intestinal flora of mice. Figure S2. LDA analysis of Lufeng aromatic vinegar on intestinal flora of mice. Table S1. Animal experimental groups. Table S2. Results of Alpha diversity index of intestinal flora in mice induced by Lufeng aromatic vinegar. Table S3. Secondary differential metabolites.

## Abbreviations

The following abbreviations are used in this manuscript:

LFAV	Lufeng aromatic vinegar
SCFAs	Short-chain fatty acids
GC-MS	Gas chromatography–mass spectrometry
LC-MS	Liquid chromatography–mass spectrometry
