# Supplementary material for: Investigating the Effects of Yunnan Lufeng Aromatic Vinegar Intervention on Intestinal Microbiota, SCFAs, and Metabolites in Mice Using Multi-Omics Techniques
Source: Foods. 2025 Oct 31;14(21):3747. doi: 10.3390/foods14213747 (PMC12609717; doi:10.3390/foods14213747)
Supplement: Supplementary file 1 [file foods-14-03747-s001.zip › foods-3888701-supplementary.pdf]

Supplementary Material

1.1 Supplementary Figures

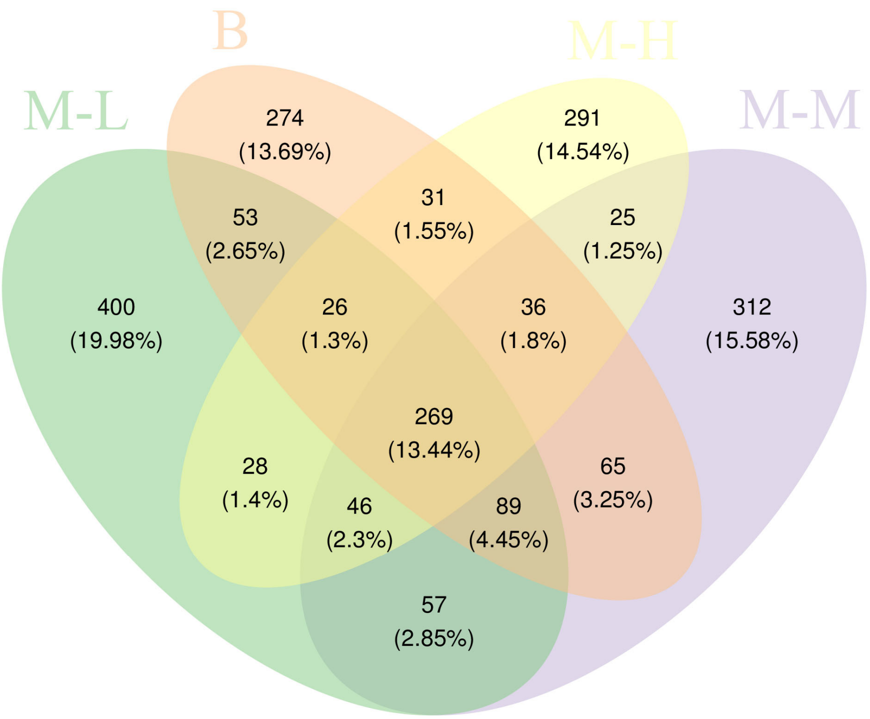

Figure S1. Venn diagram of Lufeng aromatic vinegar on intestinal flora of mice

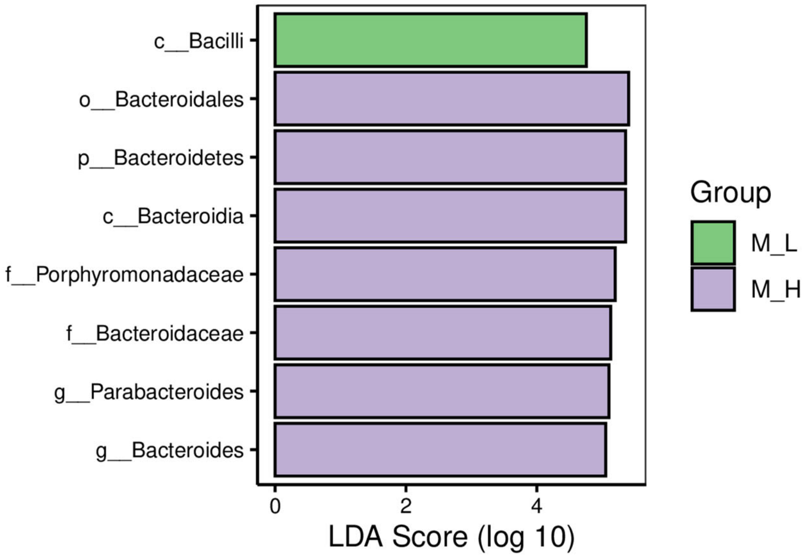

Figure S2. LDA analysis of Lufeng aromatic vinegar on intestinal flora of mice

1.2 Supplementary Table

Table S1. Animal experimental groups

Number	Group	Group name	Group specification	Size of animal	Gavage dose (ml/kg)
1	Blank control group	B	Intragastric injection of sterile water	10	-
2	Lower dose group	M-L	Intragastric injection of aging vinegar	10	3.3
3	Medium dose group	M-M	Intragastric injection of aging vinegar	10	4.95
4	High dose group	M-H	Intragastric injection of aging vinegar	10	6.6

Note: The daily gavage dose is based on the body weight of the day and is adjusted according to the experimental group design, that is, Gavage dose (ml/kg).

Table S2. Results of Alpha diversity index of intestinal flora in mice induced by Lufeng aromatic vinegar

Group	OTU	Shannon	Simpson	Chao1	Coverage
B	274	6.47±0.31[a]	0.95±0.02[a]	362.21±129.54[a]	1.00
M-L	400	6.68±0.32[a]	0.97± 0.01[a]	496.63±42.14[a]	1.00
M-M	312	6.07±0.67[a]	0.93±0.06[a]	480.52±59.36[a]	1.00
M-H	291	5.38 ±0.86[a]	0.92±0.02[a]	438.97±90.53[a]	1.00

Table S3. Secondary differential metabolites

Name	Classification	MZ	Formula	M-L	M-M	M-H	B	P.value	VIP
(2S,5S)-trans-Carboxymethylproline	Amino acid derivative	174.0759	$C_7H_{11}NO_4$	92293459.15±27.77	109169712.45±42.7	152559065.16±21.34	44087331.44±2.27	0.0029	2.36
(R)-3-Hydroxybutyric acid	Carboxylic acid	103.0396	$C_4H_8O_3$	66502115.3±8.32	40764348.81±11.12	49137669.57±21.04	87147183.52±18.86	0.0020	2.23
Sphingosine	Alcohol	300.2891	$C_{18}H_{37}NO_2$	235943062.13±18.29	208409147.87±7.89	433859630.24±37.64	141373415.92±19.14	0.0065	2.17
Melibiose	Sugar	341.109	$C_{12}H_{22}O_{11}$	16385052.77±31.21	23339304.33±23.13	10442700.27±54.01	39830202.29±17.24	0.0107	2.15
Antibiotic JI-20A	Antibiotic	482.2891	$C_{19}H_{39}N_5O_9$	24120642.57±12.71	16217803.93±45.86	15181723.6±36.78	50753236.03±27.67	0.0061	2.14
Sodium deoxycholate	Bile acids	414.321	$C_{24}H_{39}O_4$. Na	11640937.99±9.54	17708566.57±45.3	13378425.12±26.32	1385375.69±37.07	0.0001	2.09
Ethirimol	Heterocyclic compound	209.1536	$C_{11}H_{19}N_3O$	70589768±16.67	70833511.85±21.77	90076370.43±2.72	53082598.55±15.19	0.0254	2.06
N-Glycolylneuraminic acid	Sugar	324.0929	$C_{11}H_{19}NO_{10}$	36741576.44±32.97	49007075.09±36.84	37139752.19±6.95	11425252.58±49.26	0.0046	2.06
20-HETE	Fatty acid	303.2307	$C_{20}H_{32}O_3$	21761971.35±8.68	18129837.15±31.47	17021215.02±26.39	62826598.65±52.27	0.0059	2.04
Catechin	Flavonoid	271.0601	$C_{15}H_{14}O_6$	6943889.62±9.98	8535020.87±19.41	7101249.08±11.72	3106706.28±29.17	0.0011	2.04
Citraconic acid	Anhydride	129.0194	$C_5H_6O_4$	45971026.22±81.99	39361850.97±58.15	67114934.26±31.51	6630018.45±76.01	0.0457	2.03
Hydroxyphenyllactic acid	Carboxylic acid	183.0842	$C_9H_{10}O_4$	106113549.35±17.02	90852117.78±6.86	113891289.62±5.27	56150923.63±19.08	0.0007	2.03
4-Acetamido-2-aminobutanoic acid	Amino acid derivative	160.0966	$C_6H_{12}N_2O_3$	16497476.64±18.12	19869272.71±38.81	21408328.23±2.91	11236164.43±4.11	0.0296	2.02
(-)-Jasmonic acid	Fatty acid	209.1177	$C_{12}H_{18}O_3$	34704689.19±13.96	32021896.17±13.68	33455839.82±12.16	14065159.8±41.72	0.0035	2.00

4,5,6,7-Tetrahydroisoxazolo(5,4-c)pyridin-3-ol	Heterocyclic compound	141.0658	$C_6H_8N_2O_2$	15262470.75±18.88	14175799.42±6.09	14911200.77±18.04	28501938.78±6.91	0.0007	1.99
Palmitoylethanolamide	Amide	300.2884	$C_{18}H_{37}NO_2$	132574707.59±15.18	129173313.17±2.22	129321034.24±38.12	37148878.1±10.52	0.0008	1.97
Dibutyl phthalate	Ester	279.158	$C_{16}H_{22}O_4$	432709369.45±21.48	445546534.92±39.74	166524100.62±83.03	634380960.75±16.22	0.0212	1.96
Alpha-dimorphecolic acid	Fatty acid	279.2321	$C_{18}H_{32}O_3$	76310731.05±11.9	109169970.77±16.11	92252264.95±17.88	57824900.02±26.36	0.0126	1.89
Carboxyspermidine	Polyamine derivative	189.1593	$C_8H_{19}N_3O_2$	39676409.7±56.52	31235309.71±47.53	32793907.74±42.76	120926802.68±39.5	0.0200	1.87
Oleamide	Amide	282.2799	$C_{18}H_{35}NO$	62391011.02±27.71	48850638.44±18.97	55979331.67±4.11	94833923.71±5.91	0.0098	1.84
S-Glutathionyl-L-cysteine	Peptide	426.0994	$C_{13}H_{22}N_4O_8S_2$	15324687.17±49.65	28399646.67±17.98	27718937.63±22.1	12859485.8±18.6	0.0286	1.82
Adenosine	Nucleoside	268.1029	$C_{10}H_{13}N_5O_4$	168872881.69±30.37	86690813.36±29.89	118095029.26±32.16	9736743.36±46.04	0.0001	1.82
3,4-Dihydroxyphenylpropanoate	Carboxylic acid	165.0543	$C_9H_{10}O_4$	294109953.89±40.53	271159193.71±5.24	276961862.55±12.83	115457972.53±30.83	0.0115	1.80
L-Glutamine	Amino acid	146.0804	$C_5H_{10}N_2O_3$	40530728.95±49.88	31226395.19±36.4	39531956.6±17.67	13474928.8±6.32	0.0216	1.79
Indoleglycerol phosphate	Indole derivative	288.0733	$C_{11}H_{14}NO_6P$	37498946.26±23.33	18893885.05±115.83	28347138.1±142.62	109492708.45±20.75	0.0212	1.74
Thiamine	Amino acid	265.1113	$C_{12}H_{17}N_4OS$	429667069.77±82.41	37295446.25±24.8	109942055.64±90.99	488006875.94±34.69	0.0108	1.74
1-palmitoylglycerophosphocholine	Phospholipid	496.3445	$C_{24}H_{51}NO_7P$	166177215.52±9.42	161790657.55±36.54	188249372.75±12.27	439081475.22±25.65	0.0031	1.73
D-synephrine	Alkaloid	168.0909	$C_9H_{13}NO_2$	56331246.49±7.32	54654032.04±10.8	57724198.71±13.86	39414773.58±16.81	0.0204	1.70
(+)-Camphor	Monoterpenoids	153.1275	$C_{10}H_{16}O$	7589065.7±85.21	22215557.56±51.45	12570376.2±70.22	2057448.64±3.22	0.0133	1.69

L-4-Hydroxyphenylglycine	Amino acid derivative	167.0124	$C_8H_9NO_3$	22291638.63±52.63	10295594.6±9.53	18333609.65±40.46	42914219.98±38.2	0.0247	1.65
9-OxoODE	Fatty acid	277.2156	$C_{18}H_{30}O_3$	252760592.62±14.42	226265564.95±18.12	242438478.7±9.78	138819917.45±33.34	0.0172	1.65
Cholesterol sulfate	Sterol derivatives	465.3085	$C_{27}H_{46}O_4S$	29830611.8±21.88	32483958.87±6.23	33367135.37±31.22	56237377.4±7.17	0.0036	1.63
Triacetate lactone	Ester	127.0374	$C_6H_6O_3$	50410225.71±19.48	46131289.57±20.66	48853689.98±16.02	28452297.66±31.25	0.0346	1.58
Undecanoic acid	Carboxylic acid	186.9544	$C_{11}H_{22}O_2$	14779688.43±44.82	12629659.67±17.97	13728589.91±22.6	6841081.54±11.35	0.0320	1.55
Thyrotropin releasing hormone	Peptide	362.3244	$C_{16}H_{22}N_6O_4$	49247693.57±15.83	32985297.17±11.75	40403797.5±4.97	19499382.42±14.92	0.0001	1.54
L-Fucose	Sugar	165.1136	$C_6H_{12}O_5$	2861106.11±40.36	2300023.87±10.62	2599930.85±30.69	1218651.71±14.35	0.0224	1.51
Methyl (indol-3-yl)acetate	Indole derivative	172.0713	$C_{11}H_{11}NO_2$	12943274.15±7.24	22603948.69±6.86	24233961.97±8	18382161.74±23.23	0.0019	1.49
O-Phosphoethanolamine	Amino acid derivative	141.9577	$C_2H_8NO_4P$	39042100.89±5.32	43678745.69±13.38	40591683.38±5.92	53242685.35±11.01	0.0174	1.48
2,3-Dinor-8-iso prostaglandin F2alpha	Prostaglandin	309.2049	$C_{18}H_{30}O_5$	10071345.35±18.69	6053685.53±17.3	10063574.43±47.54	4170684.82±47.87	0.0375	1.45
Glycyrrhetinate	Triterpenoid	471.3511	$C_{30}H_{46}O_4$	819582.56±70.26	1603353.27±66.66	889647.4±85.87	173729.76±43.46	0.0463	1.41
(S)-Abscisic acid	Terpenoid	247.1287	$C_{15}H_{20}O_4$	5527849.57±35.79	4611449.53±64.12	4536578.42±9.76	1691716.25±28.02	0.0470	1.41
Lovastatin	Statins	404.2453	$C_{24}H_{36}O_5$	14527768.56±65.36	30082083.44±68.87	13557021.47±127.79	825947.29±94.24	0.0227	1.38
Mesaconate	Carboxylic acid	130.0168	$C_5H_6O_4$	157317540.07±39.69	109659463.93±37.03	108641699.49±29.84	41155500.64±40.99	0.0099	1.36

Xanthine	Purine bases	153.0405	$C_5H_4N_4O_2$	1405503452.07±4.94	1642497731.64±3.59	1499155004.01±26.81	1162260493.06±8.92	0.0165	1.31
4-Guanidinobutanoic acid	Amino acid derivative	146.0921	$C_5H_{11}N_3O_2$	218084769.1±23.27	245623480.98±4.02	255616492.7±37.33	394024060±2.69	0.0007	1.30
Docosapentaenoic acid (22n-3)	Fatty acid	330.2522	$C_{22}H_{34}O_2$	111879329.68±5.26	100909979.46±6.31	128830783.03±18.51	158241603.41±17	0.0157	1.28
Guanidoacetic acid	Amino acid derivative	116.9274	$C_3H_7N_3O_2$	298596199.32±4.35	334573993.96±8.1	263509165.93±5.33	314490564.34±4.15	0.0057	1.28
Cholesterol	Sterol	369.3501	$C_{27}H_{46}O$	4945877.16±12.5	2792881.06±4.42	4735997.82±24.14	2066444.71±77.26	0.0152	1.25
Anandamide	Alkaloid	348.2876	$C_{22}H_{37}NO_2$	13469348.02±13.11	24834393.93±14.72	12364072.47±14.26	9046939.45±14.09	0.0002	1.23
Oxypurinol	Purine bases	151.0257	$C_5H_4N_4O_2$	24953140.77±11.77	17623198.99±11.16	21452901.33±10.99	24154169.2±1.74	0.0106	1.22
S-Methyl-L-methionine	Amino acid	164.0738	$C_6H_{14}NO_2S$	7562472.94±40.78	6731479.77±12.39	12265935.17±16.97	7026070.87±96.6	0.0483	1.21
N-Acetylserotonin	Indole derivative	199.1704	$C_{12}H_{14}N_2O_2$	18698296.53±30.7	18343611.61±34.43	32241093.8±44.69	75633559.5±56.86	0.0287	1.20
D-Gulono-1,4-lactone	Sugar	177.0404	$C_6H_{10}O_6$	90005973.46±32.35	48247019.41±5.33	58461209.34±10.26	67670701.88±8.03	0.0185	1.16
5-Hydroxymethyluracil	Nucleoside analog	142.0345	$C_5H_6N_2O_3$	9504793.05±2.4	8608286.5±33.57	8870874.84±11.18	6772165.98±8.88	0.0331	1.15
N-a-Acetylcitrulline	Amino acid derivative	217.1073	$C_8H_{15}N_3O_4$	3996545.83±4.42	4254520.09±3.34	3850589.75±6.1	3613461.01±5.77	0.0226	1.15
N-Nitroso-pyrrolidine	Heterocyclic compound	101.071	$C_4H_8N_2O$	134177436.54±25	45679554.97±56.56	111862658.39±26.6	31016877.52±5.81	0.0045	1.15

Norlinolenic acid	Fatty acid	265.2162	$C_{17}H_{28}O_2$	3061890.87±11.72	3196854.42±9.4	2572834.49±12.36	2039469.05±26.96	0.0257	1.13
Riboflavin reduced	Vitamin	378.1482	$C_{17}H_{22}N_4O_6$	8700694.19±13.03	20664629.8±99.45	24648755.91±91.98	77131709.97±35.9	0.0087	1.10
Andrographolide	Diterpenoid compound	333.2067	$C_{20}H_{30}O_5$	50203453.67±31.41	41254594.4±11.67	33139179.89±34.97	19838065.5±32.57	0.0237	1.04
ATP	Nucleoside triphosphate	505.983	$C_{10}H_{16}N_5O_{13}P_3$	10459219.41±12.5	12901508.92±74.84	16529672.14±70.79	28316149.94±14.43	0.0070	1.03
